# Lemon Grass Essential Oil does not Modulate Cancer Cells Multidrug Resistance by Citral—Its Dominant and Strongly Antimicrobial Compound

**DOI:** 10.3390/foods9050585

**Published:** 2020-05-05

**Authors:** Jitka Viktorová, Michal Stupák, Kateřina Řehořová, Simona Dobiasová, Lan Hoang, Jana Hajšlová, Tran Van Thanh, Le Van Tri, Nguyen Van Tuan, Tomáš Ruml

**Affiliations:** 1 Department of Biochemistry and Microbiology UCT Prague, Faculty of Food and Biochemical Technology, Technicka 3, Prague 166 28, Czech Republic; prokesoj@vscht.cz (J.V.); rehorova@vscht.cz (K.Ř.); dobiasos@vscht.cz (S.D.); hoangl@vscht.cz (L.H.); 2 Department of Food Analysis and Nutrition UCT Prague, Faculty of Food and Biochemical Technology, Technicka 3, Prague 166 28, Czech Republic; stupakm@vscht.cz (M.S.); hajslovj@vscht.cz (J.H.); 3 Vietnam University of Traditional Medicine, No. 2—Tran Phu, Ha Dong, Hanoi 100000, Vietnam; thanhtv63@gmail.com; 4 Vietnam Essential Oil Joint Stock Company, No. 814/3-Lang street, Dong Da, Hanoi 100000, Vietnam; biotech.jsc@gmail.com (L.V.T.); tuanbiotech.jsc@gmail.com (N.V.T.)

**Keywords:** multidrug resistance, doxorubicin, MRSA, quorum sensing, biofilm

## Abstract

With strong antimicrobial properties, citral has been repeatedly reported to be the dominant component of lemongrass essential oil. Here, we report on a comparison of the antimicrobial and anticancer activity of citral and lemongrass essential oil. The lemongrass essential oil was prepared by the vacuum distillation of fresh *Cymbopogon* leaves, with a yield of 0.5% (*w/w*). Citral content was measured by gas chromatography/high-resolution mass spectrometry (GC-HRMS) and determined to be 63%. Antimicrobial activity was tested by the broth dilution method, showing strong activity against all tested bacteria and fungi. Citral was up to 100 times more active than the lemongrass essential oil. Similarly, both citral and essential oils inhibited bacterial communication and adhesion during *P. aeruginosa* and *S. aureus* biofilm formation; however, the biofilm prevention activity of citral was significantly higher. Both the essential oil and citral disrupted the maturated *P. aeruginosa* biofilm with the IC_50_ 7.3 ± 0.4 and 0.1 ± 0.01 mL/L, respectively. Although it may seem that the citral is the main biologically active compound of lemongrass essential oil and the accompanying components have instead antagonistic effects, we determined that the lemongrass essential oil-sensitized methicillin-resistant *S. aureus* (MRSA) and doxorubicin-resistant ovarian carcinoma cells and that this activity was not caused by citral. A 1 mL/L dose of oil-sensitized MRSA to methicillin up to 9.6 times and a dose of 10 µL/L-sensitized ovarian carcinoma to doxorubicin up to 1.8 times. The mode of multidrug resistance modulation could be due to P-glycoprotein efflux pump inhibition. Therefore, the natural mixture of compounds present in the lemongrass essential oil provides beneficial effects and its direct use may be preferred to its use as a template for citral isolation.

## 1. Introduction

*Cymbopogon citratus* (monocotyledonous plant belonging to Poaceae family), known as lemongrass, is widely used for its characteristic lemon odor and flavor in the culinary industry [1] as a component of spices. Moreover, it is used for its curative effects in traditional and alternative medicine in Asia, Africa and Latin America. Lemongrass is usually used in the form of a concoction of the aboveground parts of the plant or as an essential oil. In addition to vitamins (esp. A, C, folate, niacin, β-carotene, source: USDA National Nutrient data base), *C. citratus* contains flavonoids, alkaloids, tannins, phenols and saponins as beneficial compounds [2]. The chemical composition of the essential oil varies depending on the geographical origin of the plant. However, the main component is always citral (65%–85%), a mixture of isomers of geranial (citral a) and neral (citral b). It also contains citronellol, citronellal, limonene, linalool, nerol, etc. [3]. Lemongrass in traditional medicine is used for the treatment of colds, influenza, cough, diabetes, malaria [4], high blood pressure, high cholesterol, fever, inflammation, hypertension [5,6], dental hygiene [7], colorectal cancer [8], nervousness, toothache and sore throat [9].

The main demonstrated bioactivities are antimicrobial, anti-inflammatory, anticancer, antimutagenic and antidiabetic activities [2]. The antimicrobial activity is the most explained. Lemongrass essential oil has a nonselective activity against both Gram-negative (*Escherichia coli*, *Klebsiella pneumoniae*, *Pseudomonas aeruginosa*, *Proteus vulgaris*) and Gram-positive bacteria (*Bacillus subtilis*, *Staphylococcus aureus*), yeasts and fungi [2]. The oil was active against *Cronobacter sakazakii*, a food-borne pathogen associated with neonatal necrotizing enterocolitis. Its action is ascribed to decreasing quorum sensing, biofilm formation and endotoxin production [10]. The inhibition of sulfate-reducing bacteria, causing several issues in the petroleum industry, was demonstrated as well [11,12]. Recently, the inhibition of *Candida albicans* and *Cryptococcus* sp. biofilm formation and its disruption by the essential oil were published [13,14,15]. The activity of lemongrass oil against *Aspergillus flavus* was even higher than that of some synthetic fungicides, e.g., benzimidazole and diphenylamine [16]. Besides antimicrobial activity, an effect was also demonstrated against protozoa (*Plasmodium* sp., *Leishmania* sp.) [17,18] and insects (*Anopheles* sp., *Musca domestica*, *Aedes aegypti*) [19,20,21]. In addition, the essential oil of lemongrass is an effective repellent [22].

Despite showing strong antimicrobial activity and potency to treat diseases of human civilization, neither the ability of lemongrass essential oil nor citral to modulate drug resistance has ever been tested. Drug resistance is a major challenge for contemporary medicine. Antibiotic resistance is currently a frequent and typical complication of the treatment of diseases or injury. The first antibiotic, penicillin, was only introduced a few years before bacteria resistant to it were isolated. Similarly, antineoplastic resistance is a major challenge for treatment and for patient survival overall. Numerous generations of compounds overcoming drug resistance in therapy have been described; however, the usage of natural compounds still looks to be the most promising [23].

Although the effects of *C. citratus* extracts and especially its essential oil are studied relatively often, there are many areas that are controversial or deserve deeper exploration. In this paper, we report on the large spectrum of antimicrobial activities resulting from the chemical composition of lemongrass essential oil. Some of the activities have been already previously identified, however, we seek to provide deeper understanding and report some new explanations and context especially in the field of drug resistance modulation.

## 2. Materials and Methods

### 2.1. Chemicals

Mueller Hinton Broth 2 (MH broth, Sigma-Aldrich, St. Louis, MO, USA), Malt extract broth (ME broth, Oxoid, Hampshire, UK), Brain Heart Infusion Broth (BHI, Sigma-Aldrich), resazurin (Sigma-Aldrich), methicillin (Sigma-Aldrich), vancomycin (Sigma-Aldrich), citral (Sigma-Aldrich), casamino acids (Sigma-Aldrich), L-arginine (Sigma-Aldrich), Pgp-Glo Assay System (Promega, Madison, WI, USA), Na_3_VO_4_ (Promega), Dulbecco’s Modified Eagle’s Medium (DMEM, Sigma-Aldrich), doxorubicin (Sigma-Aldrich).

### 2.2. Plant Material and Essential Oil Extraction

*Cymbopogon citratus* (DC.) Stapf (family: Poaceae) was identified by the Viet Nam Institute of Medicinal Plants under the Ministry of Health (3 Quang Trung Str., Hoan Kiem Distr, Ha Noi, Viet Nam). The planting location was the Phu Thanh, Hung Thi and An Binh Communes, Lac Thuy District, Hoa Binh province, Mountainous North of Viet Nam. The plants were harvested during October and November 2018. The water content of the material before oil extraction was 50%. The water content was determined by weighing before and after drying.

The essential oil was distilled from the above-ground part of the plant by steam distillation under vacuum (0.5 atm, Shanghai EVP Vacuum Technology, China) at a temperature of 80–90 °C for two hours. By this procedure, which was modified according to [24], 1 kg of essential oil was obtained from 200 kg of fresh leaves. The yield of oil extraction was calculated as the percentage of obtained oil weight to fresh leaves weight. Density was determined gravimetrically.

### 2.3. Essential Oil Composition Screening

For the analysis of volatile and semi–volatile compounds in the lemongrass essential oil, gas chromatography coupled to high-resolution mass spectrometry (GC–HRMS) was employed as previously described by [25].

#### 2.3.1. Sample Preparation

Fifty grams of the lemongrass essential oil was weighted into a 15 mL polypropylene centrifuge tube and 10 mL of ethyl acetate was added. One milliliter of ethyl acetate extract was diluted in 9 mL of isooctane. Finally, 2 µl of the isooctane extract was injected into the GC-HRMS instrumentation (Agilent Technologies, Santa Clara, CA, USA).

#### 2.3.2. GC-HRMS

An Agilent 7200b system consisting of an Agilent 7890B gas chromatograph equipped with a quadrupole–time of flight mass spectrometer (Q–TOF) (Agilent Technologies, Santa Clara, CA, USA) was used for the instrumental analysis.

Sample components were separated in a 30 m HP-5MS capillary column (0.25 mm id, film thickness: 0.25 µm; Agilent Technologies, Santa Clara, CA, USA). The sample was injected in split mode (1:5) at 250 °C and the oven temperature was as follows: 40 °C (1 min), 10 °C/min to 150 °C, 5 °C/min to 190 °C, 20 °C/min to 310 °C (hold 5 min).

The mass spectrometric detector was operated in electron ionization mode and the temperature of the ion source was 230 °C. The mass range was 40–550 *m/z* and the resolution of the mass analyzer was set to >12 500 (FWHM).

Volatile and semi–volatile compounds were identified and verified by NIST library 2017, isotopic pattern, exact mass (mass error <5 ppm) and Kovats retention index.

### 2.4. Antimicrobial Activity

The inhibitory activity of the essential oil and citral was determined against both Gram-positive and Gram-negative bacterial strains, yeasts and micromycetes. Unless otherwise stated, microorganisms were obtained from the Collection of the Department of Biochemistry and Microbiology (DBM, UCT Prague, Prague, Czech Republic) as follows: *Salmonella enterica* (CCM, 4420), *Proteus vulgaris* (DBM, 3022), *Mycobacterium smegmatis* (ATCC, 70084), *Pseudomonas aeruginosa* (CCM, 3955), *Staphylococcus aureus* (ATCC, 25923), *Candida famata* (DBM, 23), *Candida albicans* (DBM, 2186), *Cryptococcus albidus* (DBM, 4), methicillin-resistant *Staphylococcus aureus* (DBM, 12). The resistant strain was obtained from the Collection of the Laboratory of Medical Microbiology (Czech Laboratory, lnc., NEM 449) and was previously characterized for its multidrug resistance properties. Unless otherwise stated, all strains are sensitive to commercial drug strains according to EUCAST (The European Committee on Antimicrobial Susceptibility Testing. Breakpoint tables for interpretation of MICs and zone diameters. Version 10.0, 2020).

Antibacterial and anti-yeast activity was evaluated by the standard broth-dilution method using 96-well plates and MH broth or ME broth. Overnight microbial culture was diluted to a turbidity equal to 0.5 McFarland. After that, the standard broth microdilution method recommended by EUCAST (The European Committee on Antimicrobial Susceptibility Testing. Breakpoint tables for interpretation of MICs and zone diameters. Version 10.0, 2020) was used. The essential oil and citral were diluted with the prepared microbial culture. Subsequent binary dilution provided the range of tested concentrations from 5 to 10,000 µL/L. The positive control was the suspension of microorganisms without essential oil. Plates were incubated for 24 h at 37 or 28 °C after that, absorbance (500 nm) was recorded using a SpectraMax i3x Multi-Mode Detection Platform (Molecular Devices, San Jose, CA, USA). The turbidity of each strain was measured in eight replicates. The turbidity of the methicillin-resistant *S. aureus* was measured in eight replicates, both with and without chloramphenicol (0.2–200 mg/L), and both with and without lemongrass essential oil.

### 2.5. Anti-Biofilm Activity

The effect of lemongrass essential oil and citral on the bacterial biofilm was tested on *Staphylococcus aureus* (ATCC, 25923) and *Pseudomonas aeruginosa* (CCM, 3955). A static anti-biofilm assay was performed in 96-well polystyrene plates, as described previously with some modifications [26]. Both samples were evaluated for their activity in two main stages (i) planktonic cell adhesion and (ii) mature biofilm disruption. The overnight culture of the tested organism was diluted with BHI broth to obtain a turbidity equal to 0.5 McFarland, and 100 µl of the suspension was pipetted into each well. In the cell adhesion assay, the viability of adhered cells was evaluated by resazurin assay immediately after 24 h of incubation in the presence of the tested compounds at 37 °C and triple washing with PBS (pH 7.4). In the disruption of mature biofilm assay, fresh BHI medium containing various concentrations of lemongrass or citral was added to wells with a pre-formed biofilm. After 24 h of incubation, the medium was removed, the wells were washed three times with PBS and 100 µl of resazurin in PBS (0.03 mg/L) was added. The viability was evaluated by measuring fluorescence (560/590 nm, ex./em.) using a SpectraMax i3x Multi-Mode Detection Platform (Molecular Devices, USA). Each experiment was done in 16 repetitions.

### 2.6. Anti-Quorum Sensing Activity

For the evaluation of anti- quorum sensing activity, two commercial (ATCC) strains of *Vibrio campbellii* were used - BAA1118 and BAA1119 [27]. The bacteria were cultivated in Autoinducer Bioassay (AB-A) medium as described previously by [28]. The overnight culture was diluted with AB-A medium to a cell density of approximately 1 × 10^4^ CFU/mL. The binary dilution of citral and lemongrass oil (0.1 µL/L—6 mL/L) was tested using *Vibrio campbellii* in a 96-well plate and after 24 h, the cell viability was determined by resazurin assay. IC_10_ was chosen for the subsequent experiment. The lemongrass essential oil or citral were applied at the IC_10_ concentration and further binary diluted with diluted cell culture. After that, the luminescence was recorded for 24 h with a measurement step of 20 min using a microplate reader (SpectraMax i3 Multi-Mode Detection Platform, Molecular Devices, San Jose, CA, USA) set to 30 °C; integration time of 10,000 ms; shaking for 60 s prior to measurement. The sum of luminescence was calculated and used for the determination of EC_50_.

### 2.7. Inhibition of Transmembrane Efflux Pump

The inhibition of P-glycoprotein (P-gp) was tested using the in vitro Pgp-Glo Assay System according to the standard procedure [29] with only slight modification of time incubation. The incubation was prolonged in our case to 60 min. The luminescence (ΔRLU samples) was calculated as the difference between the relative luminescence of Na_3_VO_4_ and that of the samples. Orthovanadate was used as a known inhibitor of P-gp, Verapamil was used as a known activator of P-gp.

### 2.8. Sensitization of Doxorubicin-Resistant Human Ovarian Carcinoma

A human ovarian carcinoma cell line (HOC, A2780) and its doxorubicin-resistant sub-line (HOC/DOX, A2780/ADR) were purchased from Sigma-Aldrich (USA). Both cell lines were cultivated as described previously [29]. For the experiment, 1 × 10^5^ cells/mL were seeded into the 96-well plates. After 24 h, the cells were washed 3× with PBS and fresh DMEM supplemented with lemongrass essential oil (10 µL/L) was added. Doxorubicin in the concentration range of 0.3–80 µM was then applied. The cell viability was evaluated after 72 h by resazurin assay as described previously [30]. The fold change was calculated as the ratio of IC_50_ for doxorubicin and IC_50_ for the doxorubicin co-treated with the lemongrass essential oil.

### 2.9. Data Processing and Statistical Analysis

The experiment was done with the appropriate number (n) of repetitions. The relative activity was evaluated as a percentage according to the formula: 100*(slope of sample fluorescence—average slope of PC)/(average slope of NC—average slope of PC). As the positive control (PC), non-treated reaction was used. As the negative control (NC), the blank reaction was used. IC_50_ values were determined using the software GraphPad Prism 7 and its function of nonlinear regression (Y = Bottom + (Top-Bottom)/(1 + 10^((LogIC-X)*HillSlope)). MIC was determined as IC_90_ using an online tool freely provided by AAT BioQuest. The data are presented as the averages of the repetitions with the standard error of the mean (SEM). Statistical significance was checked with the Excel t-test function (two-tailed distribution, heteroscedastic type). One-way analysis of variance (ANOVA) was used followed by Duncan’s post hoc test (*P* < 0.05) to show the differences between the groups. For ANOVA, the software Statistica version 12 was used (Tibco Software Inc., Palo Alto, CA, USA).

## 3. Results

The *Cymbopogon citratus* essential oil was obtained by vacuum steam distillation at 80–90 °C. The procedure yielded 0.5% (*w/w*) of the oil with the density 0.891 + 0.004 g/mL.

### 3.1. Essential Oil Composition Screening

**Figure 1:**
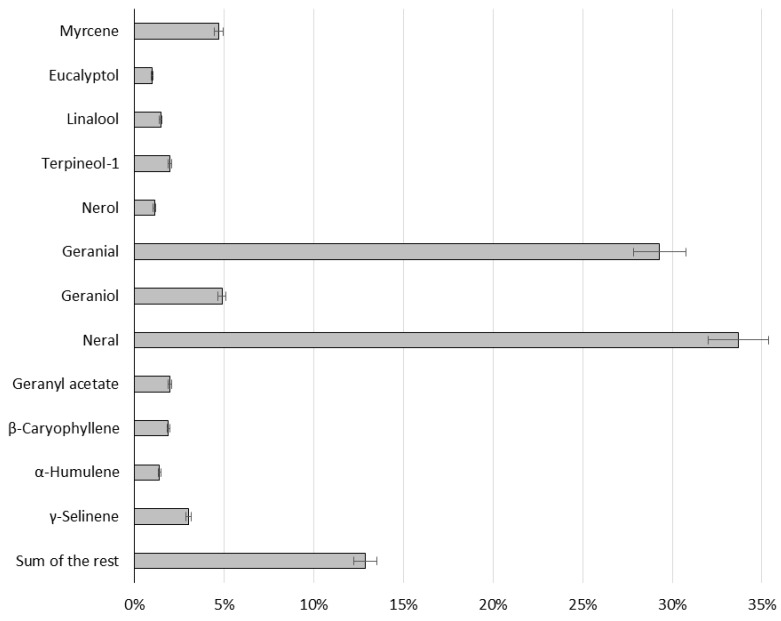
Chemical composition (%) of essential oil determined by GC–HRMS. Data are presented as an average of six repetitions (*n*) ± relative standard deviation (RSD).

The composition of the essential oil (volatile and semi–volatile compounds) was characterized by GC–HRMS. To characterize the relative abundance of volatile components in the sample, deconvolution of all compounds was performed, and the detected compounds were normalized by percent of total peaks area (Figure 1). The compounds occurring at a level higher than 1% were identified. The most dominant components in the sample were as follows: two stereoisomers of citral (geranial and neral) >> geraniol–myrcene > γ-selinene > terpineol-1–geranyl acetate > β-caryophyllene > linalool > α-humulene > nerol > eucalyptol. The repeatability of the method, expressed as a relative standard deviation (RSD), was determined by the analysis of the oil sample in six repetitions, and ranged from 1.5% to 5.5% in all analyses.

### 3.2. Antimicrobial Activity

The antimicrobial activity of lemongrass essential oil was previously published with a special focus on its strong antifungal activity. In this paper, we summarize the spectrum of its antimicrobial activity, taking into account both Gram-positive and Gram-negative bacteria and yeasts (Table 1). In agreement with previous papers, its antifungal activity was higher than its antibacterial activity. The essential oil was the most active against *C. albidus,* which could cause ocular and systematic diseases in immunosuppressed patients and against other human/animal (*C. famata*, *C. albicans*) fungal pathogens, with an IC_50_ in the concentration range of 180–570 µL/L. This antibacterial activity was shown against both Gram-positive and Gram-negative bacteria. The essential oil was also active against *M. smegmatis*, the model organism for studies of *M. tuberculosis* and other mycobacterial pathogens. Among the tested organisms, the lowest activity was determined against the Gram-negative bacteria *P. aeruginosa and S. enterica* with an IC_50_ equal to 2.4–2.6 mL/L and an MIC of almost double that. In comparison to lemongrass essential oil, citral—the dominant oil component, exhibited an IC_50_ that was several times lower with no antimicrobial specificity. In contrast to the oil, it effectively inhibited the growth of both types of bacteria and yeast in the same concentration range. Similar to the oil, citral was the most active against *C. albidus*; however, its IC_50_ was 100 × lower than the IC_50_ of the oil. Similar results were observed for the other tested microorganisms, showing that citral is the main antimicrobial agent of oil and that its activity is antagonistically modulated by the accompanying components.

The antimicrobial activity of the essential oil was also tested against antibiotic-resistant bacteria. The clinical isolate of methicillin-resistant *S. aureus* (MRSA) used in this study was characterized as multidrug-resistant to a broad spectrum of antibiotics (data not shown). The IC_50_ concentration of chloramphenicol for MRSA was 142 mg/L. To evaluate the MDR-modulating activity, MRSA was cultivated in the presence of lemongrass essential oil (1000 µL/L) or citral (400 µL/L) and chloramphenicol. Even though the citral content in the oil is 63%, this concentration (630 µL/L) was not applied because of the high toxicity of pure citral to the cells, as shown above for drug-sensitive *S. aureus* (IC_50_ 77 µL/L). Therefore, we chose concentrations of lemongrass essential oil or citral equal to IC_10_, which were applied simultaneously with chloramphenicol to determine the IC_50_ of the mixture (Table 2). The addition of lemongrass oil reduced the IC_50_ of MRSA almost tenfold (to 15 mg/L). The effect of citral on MRSA was almost three times lower, the sensitization of MRSA was only 3-fold.

### 3.3. Anti-Biofilm Activity

**Figure 2:**
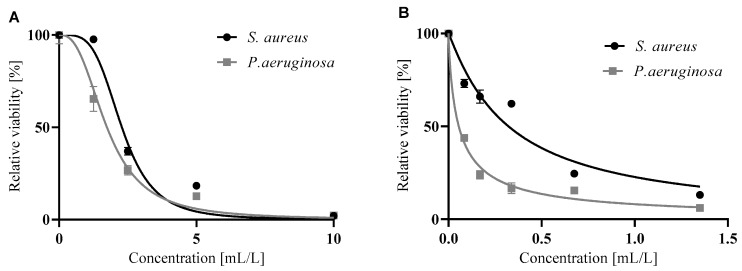
Inhibition of adhesion of bacteria forming a biofilm by lemongrass essential oil (**A**) and citral (**B**). The data are presented as an average of 16 repetitions with SEM.

**Figure 3:**
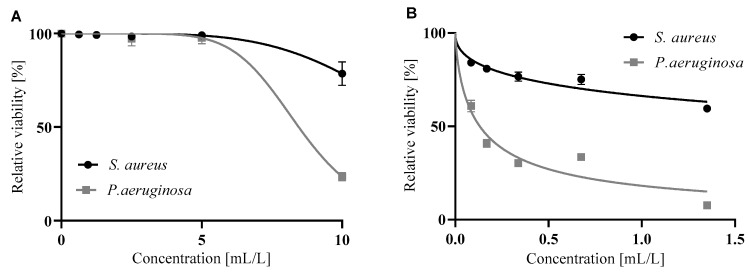
Disruption of maturated biofilm by lemongrass essential oil (**A**) and citral (**B**). The data are presented as an average of 16 repetitions with SEM.

Biofilm formation plays a crucial role in many medical and industrial applications. Typically, the effect of anti-biofilm compounds can be seen in two main stages: a) adhesion of the planktonic cells to the surface and b) disruption of a maturated biofilm. Both citral and lemongrass essential oil inhibited the adhesion of Gram-positive (*S. aureus*) and Gram-negative (*P. aeruginosa*) bacteria in a dose-dependent manner. In contrast to direct antimicrobial activity, a higher effect was observed for the adhesion of *P. aeruginosa* than for *S. aureus* (Figure 2). In the eradication of mature biofilms, just only citral disrupted bacterial biofilms in concentration-dependent manner with significantly higher effect on gram negative bacteria (*P. aeruginosa*) while lemon grass essential oil was not considered significant (Figure 3). Both citral and lemongrass essential oil disrupted the biofilm in a concentration-dependent manner with a significantly higher effect on Gram-negative bacteria (*P. aeruginosa*).

As can be seen from Table 3, which compares the concentrations of citral and lemongrass essential oil that halve the respective activity, citral was more active against both phases of biofilm formation—adhering and maturated cells. As the IC_50_ values of citral were 7–70 × lower than the IC_50_ of lemongrass essential oil, it could be concluded that citral is responsible for the main activity of the essential oil against a biofilm. However, this activity is negated by the antagonistic effect of some other compounds that are also in the oil. Citral is up to 5 × more active against the adhesion phase of *P. aeruginosa* biofilm than against an *S. aureus* biofilm.

### 3.4. Inhibition of Bacterial Cell-To-Cell Communication

**Figure 4:**
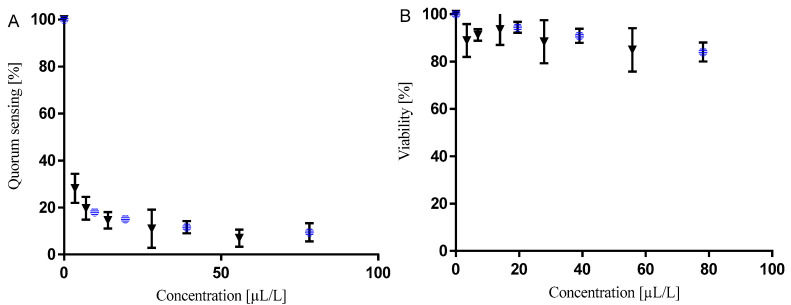
Dose-dependent effect of lemongrass essential oil (blue circle) and citral (black triangle) on *V. campbellii* BAA1118: (**A**) quorum sensing inhibition, (**B**) cell viability. The data are presented as an average of 3 repetitions with SEM.

The inhibition of bacterial quorum sensing was measured by using the sensor system of *V. campbellii*. First, the direct toxicity of the oil and citral was determined in order to avoid false-positive results. Table 4 shows the concentrations of citral and lemongrass essential oil that halved the bacterial quorum sensing and viability of mutant sensor strains of *V. campbellii*, which responds either only to (i) AI-1 autoinducer (BAA1118) or (ii) AI-2 autoinducer (BAA1119). As can be seen from the table, the concentrations that halve viability [mL/L] and those that halved communication [µL/L] differ by a factor of 1000. The decrease in luminescence was therefore caused by an inhibition of communication rather than inhibition of cell growth (Figure 4). The lemongrass essential oil inhibited communication based on both autoinducer 1 (AI-1) and autoinducer 2 (AI-2) systems similarly to citral. However, in comparison to the oil, citral effectively inhibited both systems, with a slightly higher activity for the first one. Although the oil inhibited the AI-2-mediated communication, which is based on boron compounds and used by many Gram-negative and Gram-positive bacteria, its activity was significantly higher against AI-1, which is based on homoserine lactones used by Gram-negative bacteria.

### 3.5. Modulation of MDR in Cancer Cells

**Figure 5:**
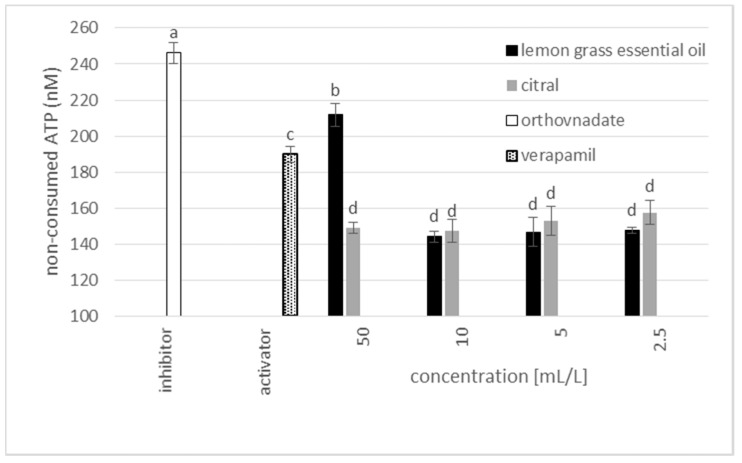
P-glycoprotein activity modulation. Orthovanadate was used as a known inhibitor of P-gp, Verapamil was used as a known activator of P-gp.

As was shown with the bacteria, lemongrass essential oil affected the multidrug resistance phenotype of MRSA. One of the main mechanisms involved in both bacteria and cancer drug-resistance is the overexpression of transmembrane efflux pumps, which transport the drug outside the cells and thus decrease its intracellular concentration [23]. Therefore, the activity of both citral and lemongrass essential oil was tested on a fraction of isolated membranes containing human P-glycoprotein (P-gp)—a transmembrane efflux pump responsible for most multidrug resistance phenotypes in tumors. As can be seen from Figure 5, lemongrass essential oil inhibited this pump. The function of P-gp is connected to the ATP consumption; therefore, the better the inhibitor, the lower the amount of consumed ATP. Compared with lemongrass essential oil, citral was unable to inhibit this pump even at higher concentration levels. Rather than being an inhibitor, citral was potentially the substrate of this pump, showing the typical trend, i.e., the higher the concentration, the higher the amount of consumed ATP.

P-glycoprotein activity inhibition was determined as the amount of ATP which was not consumed during the efflux pump activation in comparison to the control. Orthovanadate (white columns), a known inhibitor of P-gp, was used as a positive control, verapamil (dotted columns) was used as known activator and lemongrass essential oil (black columns) and citral (gray columns) were tested in the concentration range 2.5–50 mL/L. Data are presented as an average of 3 repetitions (n) with SEM. Data were analyzed by one-way ANOVA with Duncan’s post hoc test (*P* > 0.05) as indicated by the superscript letters. Statistically significant levels were denoted with different letters.

**Figure 6:**
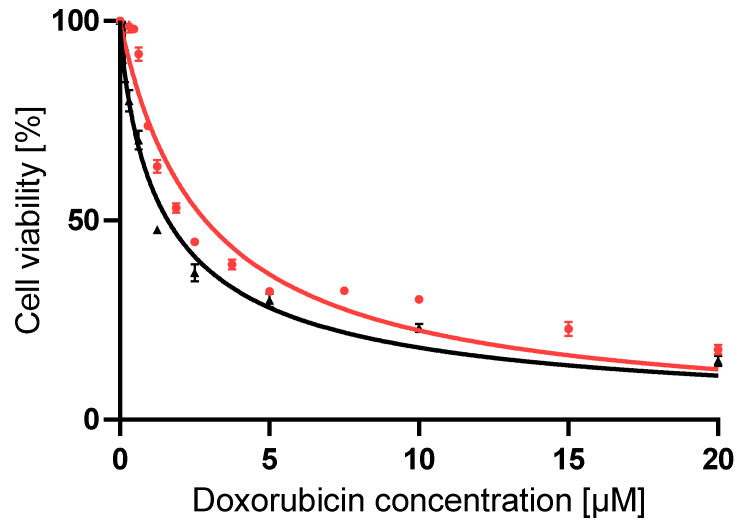
Inhibition of human ovarian carcinoma viability by doxorubicin (red line) and doxorubicin with lemongrass essential oil (10 µL/L, black line) after 72 h.

The activity of both the oil and citral were tested on an ovarian cancer cell line resistant to doxorubicin. As predicted based on the P-gp inhibition results, citral exhibited no potential to modulate the resistant phenotype of this cell line. In contrast, the co-administration of doxorubicin with the oil at a concentration far below the IC_50_ significantly decreased the cell viability (Figure 6).

The sensitization of doxorubicin-resistant ovarian carcinoma was determined as the ratio of doxorubicin concentration needed to halve the cell viability with and without lemongrass essential oil (Table 5). The resistant cell line treated with lemongrass essential oil was almost twice as sensitive as without oil, with the p value of the t-test being 0.022. As can be seen from the table, the resistant cell line is more than 100× more resistant than the sensitive line. The chosen dose of lemongrass was non-toxic even for the sensitive cancer cell line (note: viability 86% ± 4%); therefore, the sensitization effect was not caused by a direct cytotoxicity of the oil. Citral at the same concentration level (10 µL/L) did not modulate the resistant phenotype and its presence did not affect the values of doxorubicin´s IC_50_.

## 4. Discussion

Although the promising biologic activities of both lemongrass essential oil and citral have been known for decades, their modulation of the drug-resistant phenotype has never been tested in as much depth as by this paper. Here we report on the modulation of both bacterial and cancer drug resistance by lemongrass essential oil; however, based on our results, this activity is not caused by citral. The activity of *Cymbopogon flexuosus* essential oil against multi-drug resistant bacteria was previously studied using *Acinetobacter baumannii* strains [25]. The mean value of MIC was about 0.65% (*v/v*). Our IC_50_ value for MRSA (0.4%, *v/v*) agrees with published data. The sensitization factor of methicillin-resistant *S. aureus* (MRSA) by lemongrass essential oil was several times higher than the factor for citral. Therefore, the oil is more promising for modulating a bacterial drug-resistance phenotype. In addition, Berdejo et al. [31] reported on an enhanced resistance in *S. aureus* after sub-inhibitory doses of citral exposure. This was in agreement with the study of Chueca et al. [32], who published that the exposure of a hyper-resistant strain of *E. coli* to a sub-inhibitory concentration of citral did not increase the direct resistance globally; however, it led to the emergence of several mutants displaying an increased minimum inhibitory concentration of citral. Therefore, the application of oil as a natural component mixture is in agreement with the current trend of drug resistance treatment based on a combination of biologically active compounds with different targets.

Both lemongrass essential oil and citral possess strong antimicrobial activity, even against antibiotic-resistant strains; therefore, their activity against a biofilm was evaluated within this paper as well. The disruption of a matured biofilm was only observed for Gram-negative bacteria treated with either citral or with the oil. The application of 0.06 mL/L of citral eliminated half of the *P. aeruginosa* cells adhesion, which is in agreement with the results of Espina et al., who demonstrated that the biofilm formation of resistant *S. aureus* was significantly inhibited by 0.2 mL/L of citral [33]. Lemongrass essential oil was previously used for an oral spray preparation intended to inhibit *Streptococcus* sp. biofilm formation [34]. Similarly, both oil and citral prevented the biofilm formation of sulfate-reducing bacteria [12]. Both papers are in agreement with our results, which show that relatively low citral doses of 61 and 323 ppm inhibit the adhesion of both Gram-negative and positive bacteria by 50%, respectively. Lemongrass essential oil inhibited the adhesion of both cell types as well, however at several times higher concentrations. In another study, 0.2 µg/mL of citral significantly inhibited mixed biofilm (*S. aureus* and *S. enterica*) formation, which could be caused by decreasing AI-2-mediated quorum sensing communication [35]. The inhibition of AI-2 production by citral treatment was demonstrated by [36]. Our results also proved that citral significantly inhibited both biofilm formation and bacterial communication via AI-2; however, the inhibition of quorum sensing based on AI-1 was more effective and to our knowledge, this is the first report on such activity.

The potential of citral to interact with transmembrane efflux pumps has not been satisfactorily explained yet. It was previously shown that citral acts neither as a P-gp inhibitor (ABCB1) nor as a multidrug resistance protein 1 and 2 (MRP1, ABCC1; MRP2, ABCC2) inhibitor [37,38,39]. Moreover, citral should not affect ATPase activity in any of these pumps [38]. On the other hand, Queiroz et al. [40] observed that citral downmodulated the activity and inhibited the expression of multidrug resistance associated protein 1 (MRP1). In contrast to lemongrass essential oil, citral up to a concentration of 50 mL/L was unable to inhibit pure P-gp in the in vitro system and did not affect the DOX sensitivity of doxorubicin-resistant ovarian carcinoma over 72 h of incubation. The resistance of the cell line is caused by P-gp overexpression [29]. Therefore, the 10-µL/L dose of citral should not affect the expression level of P-gp, as the cells preserved the drug-resistant phenotype. As in bacteria, the lemongrass essential oil, being a natural mixture of biologically active compounds, is more promising in overcoming drug resistance in eukaryotic cells.

The antimicrobial activity of both lemongrass essential oil and citral have been reported many times before, but here we compared the activity of the same essential oil and pure citral against prokaryotic and eukaryotic organisms, i.e., Gram-positive, Gram-negative bacteria and yeasts, respectively. Despite the similarity of some of our results with published data, we found some differences, e.g., in the minimal oil concentration inhibiting *C. albicans* (MIC), which was published as 288 mg of oil per liter [41]. Our determination of the MIC for *C. albicans* gave 2734 µL/L, which corresponds to an MIC of 2435 mg of oil per liter (recalculated based on the gravimetrically defined density of our oil as 0.891 ± 0.004 g/mL). The difference could be explained by the different sensitivity of yeast strains. The same reasons may apply for differences between our MIC of *C. albicans*, which about 14 times higher than that obtained by Sacchetti et al. [42].

The antimicrobial activity of citral was previously measured for *E. coli*. The determined value (≥0.01%) [43] corresponds to our observation for Gram-negative bacteria (41–97 µL/L). Similar to our results, Gupta et al. also demonstrated that citral has a stronger antimicrobial activity than lemongrass essential oil [44]. Citral was up to 10 times more active against some *Acinetobacter baumannii* strains than lemongrass essential oil in the study of Adukwu et al. [25].

The quality of the oil is generally assessed by its content of citral, high oxygenated monoterpenes, low monoterpene and sesquiterpene hydrocarbons, and finally low oxygenated sesquiterpenes. Our lemongrass essential oil had a standard composition comparable to previously published data. The yield of essential oil obtained by our distillation of fresh leaves was 0.5% (*w/w*), which corresponds to the data of other authors, who published yields of 0.6% [45] or 0.7% [46,47,48]. As expected, the dominant group of terpenes present in essential oil was comprised of oxygenated monoterpenes with the main components being neral (citral b, 33.7%) and geranial (citral a, 29.3%). The amounts of citral isomers differ in the literature, typically with a higher content of neral. The common contents are 36.2% [41], 39.5% [48], 41.3% [42], 41.8% [47], 42.2% [45], 50.5% [25], 52.9% [34], up to 56.8% [49] for citral a and 26.5% [41], 30.4% [47], 32.3% [42], 32.5% [45], 33.1 [49], 35.5% [48], 38.1% [34], up to 38.5% [25] for neral. The presence of 1.5% linalool is comparable with the data published by other authors—0.4% [49], 1.3% [42], 2.8% [25]. The level of geraniol was also similar, which we quantified as 4.9% and other research groups as 2.2%–8.2% [25,48].

## 5. Conclusions

Although the antimicrobial activity of citral is to some extent negated by the accompanying components of lemongrass essential oil, these components have promising activity against antibiotic-resistant bacteria and chemotherapeutic-resistant tumors. Citral preserves its strong antimicrobial activity, resulting in direct bacterial cytotoxicity, the inhibition of quorum sensing and influence on cell adhesion during biofilm formation as well as disruption of a matured biofilm. Further fractionation searching for bioactive compounds modulating the drug-resistance phenotype is a promising therapeutic approach.

## Figures and Tables

**Table 1:** Antimicrobial activity of lemongrass essential oil.

		Lemongrass Essential Oil		Citral	
species	Classification	IC_50_ [µL/L]	MIC [µL/L]	IC_50_ [µL/L]	MIC [µL/L]
*Candida famata*	Fungi, Ascomycota	177 ± 19 ^a^	3684 ± 271 ^c,d^	37 ± 7 ^b^	142 ± 19 ^b,c^
*Cryptococcus albidus*	Fungi, Basidiomycota	199 ± 25 ^a^	265 ± 31 ^a^	2 ± 0 ^a^	20 ± 6 ^a^
*Candida albicans*	Fungi, Ascomycota	571 ± 109 ^a,b^	2734 ± 250 ^b,c^	83 ± 8 ^d,e^	110 ± 15 ^b,c^
*Mycobacterium smegmatis*	Bacteria, Gram positive	860 ± 89 ^b^	3409 ± 775 ^c,d^	109 ± 12 ^e^	137 ± 19 ^b,c^
*Proteus vulgaris*	Bacteria, Gram negative	992 ± 37 ^b^	1453 ± 40 ^a,b^	97 ± 12 ^e^	163 ± 34 ^c^
*Staphylococcus aureus*	Bacteria, Gram positive	1841 ± 199 ^c^	5830 ± 198 ^e^	77 ± 2 ^e^	92 ± 2 ^b,c^
*Pseudomonas aeruginosa*	Bacteria, Gram negative	2385 ± 162 ^d^	5308 ± 339 ^e^	41 ± 2 ^b^	93 ± 8 ^b^
*Salmonella enterica*	Bacteria, Gram negative	2626 ± 301 ^d^	4693 ± 634 ^d,e^	66 ± 8 ^c,d^	97 ± 3 ^b,c^

The data are presented as an average of eight repetitions (*n*) ± standard error of the mean (SEM). The data were analyzed by one-way ANOVA with Duncan’s post hoc test (*P* ˃ 0.05) as indicated by the superscript letters. The letters indicate the differences between the groups within one assay. Statistically significant levels are denoted with different letters. The data are presented as the concentration (µM) that (i) halved the cell viability (IC_50_) or (ii) reduced 90% of cell viability (MIC).

**Table 2:** Sensitization of methicillin-resistant *Staphylococcus aureus* (MRSA) by lemongrass essential oil and citral.

	Chloramphenicol IC_50_ [mg/L]	Chloramphenicol IC_50_ [mg/L] Affected by Lemongrass Essential Oil [1000 µL/L]	Chloramphenicol IC_50_ [mg/L] Affected by Citral [400 µL/L]
MRSA	142 ± 10	15 ± 2	42 ± 1
Fold		9.6 ± 1.9	3.4 ± 0.3

The data are presented as an average of eight repetitions (*n*) ± standard error of the mean (SEM).

**Table 3:** Concentration of citral and lemongrass essential oil that halves the respective activity: (i) adhesion of bacteria forming biofilm and (ii) mature biofilm.

	Anti-adhesion IC_50_ [mL/L]		Anti-biofilm IC_50_ [mL/L]	
	*S. aureus*	*P. aeruginosa*	*S. aureus*	*P. aeruginosa*
**Citral**	0.32 ± 0.03	0.06 ± 0.01	>1.5	0.11 ± 0.01
**Lemongrass essential oil**	2.16 ± 0.11	1.90 ± 0.20	>10	7.34 ± 0.40

Data are presented as an average of 16 repetitions with SEM.

**Table 4:** The effect of citral and lemongrass essential oil on the quorum sensing and viability of *Vibrio campbellii*.

	*V. campbellii* BAA1118			*V. campbellii* BAA1119		
	Viability [mL/L]		QS IC_50_ [µL/L]	Viability [mL/L]		QS IC_50_ [µL/L]
	**IC_50_**	**IC_10_**		IC_50_	IC_10_	
**citral**	0.24 ± 0.11	0.09 ± 0.05	0.26 ± 0.03	0.99 ± 0.04	0.68 ± 0.08	1.7 ± 0.8
**lemongrass essential oil**	0.17 ± 0.01	0.05 ± 0.01	1.4 ± 0.03	5.42 ± 0.09	4.07 ± 0.16	259 ± 88

The data are presented as an average of 3 repetitions with SEM.

**Table 5:** Modulation of resistance to doxorubicin by lemongrass essential oil. The table shows the concentrations of doxorubicin that halved the viability of human ovarian carcinoma (HOC) cell lines in the presence of lemongrass essential oil and doxorubicin with a single-dose addition of lemongrass essential oil (10 µL/L). Both HOC and the same line resistant to doxorubicin (HOC/DOX) were tested.

	HOC	HOC/DOX
Doxorubicin, IC_50_ [µM]	0.022 ± 0.001	2.86 ± 0.18
Lemongrass, IC_50_ [µL/L]	55.2 ± 8.1	197.8 ± 5.7
Doxorubicin, IC_50_ [µM] with lemongrass essential oil [10 µL/L]		1.60 ± 0.14
Sensitization FOLD		1.78 ± 0.27

The sensitization factor was calculated as the ratio of IC_50_ [doxorubicin, µM] and IC_50_ [doxorubicin in the presence of lemongrass essential oil, µM]. The data are presented as an average of 3 repetitions with SE.
